# Safety Profile of Pimavanserin Therapy in Elderly Patients with Neurodegenerative Disease-Related Neuropsychiatric Symptoms: A Phase 3B Study

**DOI:** 10.3233/JAD-231167

**Published:** 2024-03-05

**Authors:** Gus Alva, Wiesław J. Cubała, Ana Berrio, Bruce Coate, Victor Abler, Sanjeev Pathak

**Affiliations:** a Department of Psychiatry and Neuroscience, ATP Clinical Research, University of California at Riverside, Riverside, CA, USA; b Department of Psychiatry, Faculty of Medicine, Medical University of Gdańsk, Gdańsk, Poland; c ACADIA Pharmaceuticals Inc., Princeton, NJ, USA

**Keywords:** Alzheimer’s disease, elderly patients, neurodegenerative diseases, neuropsychiatric symptoms, pimavanserin, safety

## Abstract

**Background:**

Pimavanserin, a 5-HT_2A_ receptor inverse agonist/antagonist, is the only medication approved by the FDA for the treatment of hallucinations and delusions associated with Parkinson’s disease psychosis (PDP). Further expanding knowledge of the safety profile of pimavanserin in PDP and neurodegenerative diseases (NDD) such as Alzheimer’s disease is of great interest for informing its use in patients with PDP (with or without dementia), given this population is highly sensitive to adverse effects following antipsychotic use.

**Objective:**

This trial evaluated the effects of pimavanserin compared to placebo in frail older adults and elderly patients with neuropsychiatric symptoms related to NDD, such as hallucinations and delusions, to better understand the safety of pimavanserin in this population.

**Methods:**

This was a phase 3b, 8-week treatment (study duration of up to 16 weeks), multicenter, randomized, double-blind, placebo-controlled, two-arm parallel-group trial (NCT03575052). The primary endpoint was safety and tolerability, measured by treatment-emergent adverse events (TEAEs). Secondary safety endpoints were change from baseline in motor and cognitive function; exploratory endpoints included suicidality, sleep quality, and neuropsychiatric symptoms.

**Results:**

Incidences of TEAEs were similar between treatment groups; 29.8% reported ≥1 TEAE (pimavanserin: 30.4%; placebo: 29.3%), and 1.8% reported serious TEAEs (pimavanserin: 2.0%; placebo: 1.5%). Pimavanserin did not impact motor- or cognitive-related function.

**Conclusions:**

Pimavanserin was well tolerated and not associated with motor or cognitive impairment. Together, these findings highlight the manageable and generally favorable safety profile of pimavanserin in patients with NDD, contributing to our knowledge on the safety of pimavanserin as it generalizes to patients with PDP.

## INTRODUCTION

Pimavanserin is the only medication approved by the US Food and Drug Administration (FDA) for the treatment of hallucinations and delusions associated with Parkinson’s disease psychosis (PDP) [1–3]. Pimavanserin is a selective 5-hydroxytryptamine receptor 2A (5-HT_2A_) inverse agonist and antagonist [4, 5] with no meaningful binding affinity for other G-protein coupled receptors, including dopamine receptors [4, 6]. Due to its specificity, pimavanserin is not associated with the same off-target effects commonly observed with the off-label use of atypical antipsychotics, such as sedation and impaired cognitive ability [7–10]. Because of these adverse effects, the clinical benefits of these atypical antipsychotics may be limited [11, 12].

Neuropsychiatric symptoms, such as anxiety and depression, hallucinations and delusions, apathy and agitation, and sleep disruption, are common in patients with neurodegenerative diseases (NDD) [13]. Most patients with a form of dementia will experience neuropsychiatric symptoms over the course of their disease [14–16]. These symptoms cause distress for patients and caregivers alike, as they can result in impairment of activities of daily living, cause poor health-related quality of life, and increase caregiver burden and stress [15, 17].

Frail older adults and elderly patients with NDD are a highly sensitive patient group due to their vulnerability to adverse effects associated with antipsychotic treatment, such as increased risk for falls, parkinsonism and dyskinesia, hypotension, and death [18, 19]. Additionally, this patient population is more likely to experience side effects due to existing comorbidities and complications from taking multiple medications [20, 21]. Therefore, evaluation of the effects of pimavanserin in patients with NDD is of particular importance for expanding our knowledge of the safety profile of pimavanserin in PDP (with or without dementia), as patients with NDD have overlapping symptoms with PDP and share a common mechanism of disease and underlying pathophysiology.

Here, we conducted an 8-week treatment (total study duration of up to 16 weeks), Phase 3b, multicenter, parallel-group study to assess the safety profile and efficacy of pimavanserin in a large sample of patients with NDD using a placebo-controlled paradigm. These results will help better inform the safety of treatment with pimavanserin in a sensitive population for its clinical use in patients with PDP.

## METHODS

### Eligibility

Eligibility criteria for the study included the following: 1) male or female ≥60 years of age and able to provide written informed consent themselves or through a legal representative or caregiver; 2) patient requires some or complete assistance with instrumental or basic activities of daily living; 3) patient meets clinical criteria for an NDD (including PD [with or without dementia], dementia with Lewy bodies, Alzheimer’s disease, and all-cause dementia) and has a Mini-Mental State Examination (MMSE) score≥6 and a Clinical Global Impression-Severity (CGI-S) score (a measure of neuropsychiatric symptoms) ≥4 at baseline; and 4) patient has a neuropsychiatric symptom severe enough to warrant antipsychotic treatment, as evidenced by a Neuropsychiatric Inventory (NPI) score (frequency×severity) ≥4 on at least one domain: (a) delusions, (b) hallucinations, (c) depression or dysphoria, (d) apathy/indifference, (e) disinhibition, (f) irritability or lability, and (g) sleep disorders. The main exclusion criteria for the study included the following: 1) patient with neuropsychiatric symptoms attributable to substance abuse; 2) patient in hospice or receiving end-of-life palliative care or bedridden; 3) patient with an unstable medical disorder.

### Study design

**Fig. 1:**
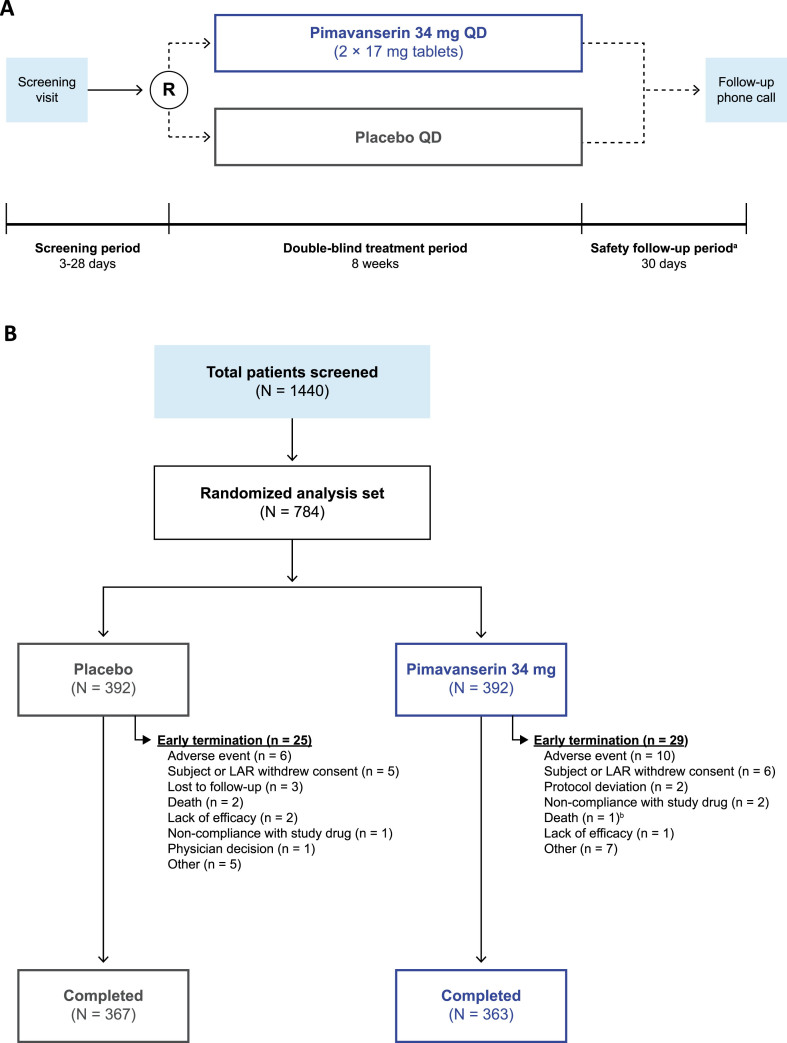
**The (A) study design and (B) patient disposition of the trial**. ^a^Subjects who enrolled in the open-label extension study did not complete the safety follow-up period. ^b^In the pimavanserin group, one patient was discontinued from the study due to an adverse event and died 4 days after the early termination visit and 4 days after stopping study drug. LAR, legally acceptable representative; R, randomization; QD, once daily.

The study design of this Phase 3b, multicenter, randomized, double-blind, placebo-controlled, parallel-group trial is represented in Fig. 1A. Prior to study initiation, all participants provided written informed consent. After screening and baseline assessments, patients were randomized (1 : 1) to receive either oral, once-daily pimavanserin (34 mg) or placebo during the double-blind treatment period (approximately 8 weeks). All patients and investigators were blinded to treatment allocation throughout the study period. Assessments were scheduled at Weeks 1, 2, 4, 6, and 8 (end of treatment). At approximately 30 (+4) days after the last dose of the study drug, patients who discontinued prematurely from the study or who did not enroll in the open-label extension study received a safety follow-up telephone call to evaluate adverse events (AEs) and posttreatment medication use. This study was approved by Advarra IRB (Columbia, MD, USA) IRB#00000971 IORG (FWA # 00023875) and performed in compliance with the Declaration of Helsinki, and study procedures were consistent with the International Council for Harmonisation/Good Clinical Practice and applicable regulatory requirements. This study was registered at clinicaltrials.gov (NCT03575052).

### Assessments

The primary endpoint for the study was safety assessed by treatment-emergent AEs (TEAEs), which were evaluated at each visit. A TEAE was defined as an AE that started on or after the first dose of the study drug and no later than 30 days after the last dose of the study drug. The secondary endpoints for safety and tolerability included the Extrapyramidal Symptom Rating Scale-Abbreviated (ESRS-A) for monitoring any worsening in extrapyramidal symptoms and the MMSE to measure any impacts on cognitive function. The MMSE was assessed at screening, baseline, and each corresponding study visit; the ESRS-A was assessed at baseline and each following study visit.

Exploratory endpoints related to the improvement of neuropsychiatric symptoms included evaluations of the potential clinical benefit of pimavanserin compared to placebo on neuropsychiatric symptoms (a change from baseline to Week 8 in the CGI-S and Clinical Global Impression-Improvement [CGI-I] scales), health outcome measures (mobility, self-care, usual activities, pain and discomfort, and anxiety and depression) using the 5-level version of the EQ-5D-5L visual analog scale, and sleep disturbances using the Sleep Disorders Inventory (SDI). Additionally, this study assessed safety, tolerability, and outcomes related to suicidality. Risk for suicidality was measured using either the Columbia-Suicide Severity Rating Scale (C-SSRS) or the Global Clinician Assessment of Suicidality (GCAS) if the patient was not able to reliably complete the C-SSRS per the study investigator’s judgment (i.e., subjects whose dementia caused them to be unable to complete the assessment reliably). All secondary and exploratory endpoints were chosen to further contribute to the safety and efficacy profile of pimavanserin for this patient population.

### Statistical analysis

Safety, tolerability, and efficacy endpoints were summarized using descriptive statistics, and TEAE percentages were summarized. Change from baseline scores were analyzed using mixed-effects model repeated measures (MMRM) except for with data collected using the EQ-5D-5L visual analog scale, which were analyzed using ANCOVA. The between-group differences in least squares mean (LSM) and corresponding standard error (SE), *p*-value were reported as difference in change from baseline values; NDD subgroups were compared for secondary and exploratory analyses.

## RESULTS

### Patient disposition and baseline characteristics

In total, 1,440 patients were screened; of these, 730 completed the study (93.1%), and 54 patients (6.9%) terminated the study early (Fig. 1B). The most common reason for study termination was AEs (*n* = 16; 2.0%), including cardiac disorders, infections, injuries, and nervous system disorders. Baseline demographics were similar between groups (Table 1). The mean (range) age was 72.4 years (60–96 years), most patients were female (57.8%), most were White (93.8%), and approximately one-third were Hispanic or Latino (31.1%). Most patients (356; 45.4%) were primarily enrolled in the 65–74-year-old age group; 31.4% were located in North America, 63.8% in Europe, and 4.8% in the rest of the world. Dementia subtypes included Alzheimer’s disease (68.4%), vascular dementia (19.3%), PD (9.2% *; patients with PD and dementia (PDD): 5.1% *; patients without dementia: 3.7%; patients with PDD and PDP: 7.8%; *note: 3 of the patients with PD and dementia also had Alzheimer’s disease, which was considered their primary cause of dementia for this analysis, therefore, values with an asterisk do not sum to the total), frontotemporal dementia (2.2%), and dementia with Lewy bodies (1.4%).

**Table 1:** Baseline demographics and clinical characteristics

	Placebo	PIM 34 mg	Total
	(*N* = 392)	(*N* = 392)	(*N* = 784)
Sex (female), *n* (%)^a^	213 (54.3)	240 (61.2)	453 (57.8)
Age, mean (SE)	72.1 (0.36)	72.7 (0.35)	72.4 (0.25)
Age categories at screening (y), *n* (%)^a^
<65	64 (16.3)	54 (13.8)	118 (15.1)
65 to 74	187 (47.7)	169 (43.1)	356 (45.4)
75 to 84	121 (30.9)	151 (38.5)	272 (34.7)
≥85	20 (5.1)	18 (4.6)	38 (4.8)
Dementia subtype, *n* (%)
Alzheimer’s disease	260 (66.3)	276 (70.4)	536 (68.4)
Vascular dementia	80 (20.4)	71 (18.1)	151 (19.3)
Parkinson’s disease^b,c^	37 (9.4)	35 (8.9)	72 (9.2)^d^
Without dementia	13 (3.3)	16 (4.1)	29 (3.7)
With dementia	24 (6.1)	16 (4.1)	40 (5.1)
Frontotemporal dementia	9 (2.3)	8 (2.0)	17 (2.2)
Dementia with Lewy bodies	6 (1.5)	5 (1.3)	11 (1.4)
Race (White), *n* (%)^a^	367 (93.6)	368 (93.9)	735 (93.8)
Ethnicity, *n* (%)^a^
Hispanic or Latino	124 (31.6)	120 (30.6)	244 (31.1)
Not Hispanic or Latino	268 (68.4)	272 (69.4)	540 (68.9)
Region, *n* (%)^a^
North America	123 (31.4)	123 (31.4)	246 (31.4)
Europe	250 (63.8)	250 (63.8)	500 (63.8)
Rest of the world	19 (4.8)	19 (4.8)	38 (4.8)
MMSE, mean (SE)	18.6 (0.23)	18.4 (0.24)	18.5 (0.17)
CGI-S, mean (SE)	4.5 (0.03)	4.6 (0.03)	4.6 (0.02)
EQ-5D-5L visual analog scale, mean (SE)	52.7 (0.92)	54.6 (0.94)	53.6 (0.66)
ESRS-A, mean (SE)	6.1 (0.56)	5.9 (0.53)	6.0 (0.38)
SDI, mean (SE)	1.3 (0.08)	1.2 (0.07)	1.2 (0.05)
QTcF, mean (SE)	407.9 (0.92)	409.5 (0.87)	–
Ever had suicidal ideation or behavior, assessed with C-SSRS or GCAS, yes, *n* (%)^a^	6 (1.7)	6 (1.6)	12 (1.7)

^a^*n* = subjects with non-missing data in the given treatment group and was used as the denominator for calculating percentages. ^b^Three subjects have Parkinson’s disease but do not have Parkinson’s disease as the primary cause of dementia. ^c^Includes Parkinson’s disease subjects with and without dementia. ^d^The proportions were balanced across pimavanserin and placebo. CGI-S, Clinical Global Impression-Severity; C-SSRS, Columbia-Suicide Severity Rating Scale; ESRS-A, Extrapyramidal Symptom Rating Scale-Abbreviated; EQ-5D-5L, 5-level version of EQ-5D-5L; GCAS, Global Clinician Assessment of Suicidality; MMSE, Mini-Mental State Examination; PIM, pimavanserin; QTcF, corrected QT interval using Fridericia’s correction method; SDI, Sleep Disturbances Inventory.

Baseline clinical characteristics were also similar between groups. The mean and standard error (SE) overall MMSE total score was 18.5 (0.17), which was similar between the pimavanserin (18.4 [0.24]) and placebo groups (18.6 [0.23]). Nearly half of patients (48.6%) scored in the 18–24 MMSE total score category. The mean (SE) CGI-S score of all patients at baseline was 4.6 (0.02), and most patients were considered either moderately (49.4%) or markedly ill (45.8%). Prior to baseline, 1.7% of patients had suicidal ideation or behavior measured using either the C-SSRS or GCAS scales.

Mean (SE) ESRS-A scores at baseline were similar between pimavanserin (5.9 [0.53]) and placebo groups (6.1 [0.56]), as were mean (SE) SDI scores (pimavanserin: 1.2 [0.07]; placebo: 1.3 [0.08]) and EQ-5D-5L visual analog scale scores (pimavanserin: 54.6 [0.94]; placebo: 52.7 [0.92]). Additionally, the baseline mean (SE) QTcF interval was 409.5 ms (0.87) in pimavanserin and 407.9 ms (0.92) in placebo groups.

### Primary endpoint: treatment-emergent adverse events

Overall, 93% of patients completed the study. A total of 234 patients (29.8%) reported experiencing at least one TEAE in the study (pimavanserin: 30.4%; placebo: 29.3%) (Table 2). Serious TEAEs were reported in 14 patients (overall: 1.8%; pimavanserin [2.0%] vs placebo [1.5%]), and TEAEs leading to discontinuation or study termination were reported in 19 patients (overall: 2.4%; pimavanserin [2.6%] vs placebo [2.3%]). The most frequently reported TEAEs included urinary tract infection (pimavanserin: 6.4%; placebo: 4.1%) and headache (pimavanserin: 2.0%; placebo: 3.8%). Four patients (0.5% in each group) had a TEAE resulting in death; none of these deaths were considered related to the study drug. Similar rates of the most common TEAEs (occurring in ≥1% of patients) were also similar between groups (Table 3).

**Table 2:** Summary of treatment-emergent adverse events

	Placebo (*N* = 392)		PIM 34 mg (*N* = 392)		Total (*N* = 784)	
	Patients, *n* (%)	Events, *n*	Patients, *n* (%)	Events, *n*	Patients, *n* (%)	Events, *n*
Any TEAE	115 (29.3)	205	119 (30.4)	220	234 (29.8)	425
Any related TEAE^a^	30 (7.7)	50	30 (7.7)	49	60 (7.7)	99
Any serious TEAE	6 (1.5)	9	8 (2.0)	11	14 (1.8)	20
Any related serious TEAE^a^	–	–	–	–	–	–
Any TEAE leading to study drug discontinuation or study termination	9 (2.3)	9	10 (2.6)	10	19 (2.4)	19
Any TEAE resulting in death^b^	2 (0.5)	2	2 (0.5)	2	4 (0.5)	4

Adverse events were coded using MedDRA version 23.0. *N* was used as the denominator for calculating percentages within each treatment group for the subject counts. A TEAE was an adverse event with onset date on or after the first study dose date and no later than the last study dose date + 30 days. Subjects may have had more than one TEAE per category. In the “Events” column, all occurrences of TEAEs were counted per category. ^a^Events with missing relationship were considered related. ^b^Included deaths that occurred within 30 days of last dose of study drug. PIM, pimavanserin; TEAE, treatment-emergent adverse event.

**Table 3:** Treatment-emergent adverse events occurring in ≥1% of patients

	Placebo (*N* = 392)		PIM 34 mg (*N* = 392)	
	Patients, *n* (%)	Events, *n*	Patients, *n* (%)	Events, *n*
Urinary tract infection	16 (4.1)	16	25 (6.4)	26
Headache	15 (3.8)	17	8 (2.0)	10
Anxiety	5 (1.3)	5	7 (1.8)	8
Blood creatine phosphokinase increased	4 (1.0)	4	5 (1.3)	5
Dizziness	3 (0.8)	6	6 (1.5)	8
Nausea	3 (0.8)	3	6 (1.5)	6
Tremor	5 (1.3)	5	4 (1.0)	4
Electrocardiogram QT prolonged	3 (0.8)	3	5 (1.3)	5
Fall	1 (0.3)	1	4 (1.0)	4
Hypertension	–	–	5 (1.3)	5

Adverse events were coded using MedDRA version 23.0. *N* was used as the denominator for calculating percentages within each treatment group for the subject counts. A TEAE was an adverse event with onset date on or after the first study dose date and no later than the last study dose date + 30 days. Subjects may have had more than one TEAE per category. In the “Events” column, all occurrences of TEAEs were counted per category. PIM, pimavanserin.

### Secondary endpoints: extrapyramidal symptoms and cognition

No significant differences were observed between groups in change from baseline to Week 8 in extrapyramidal symptoms measured using the ESRS-A (LSM [SE]: pimavanserin, −0.5 [0.19]; placebo, −0.6 [0.19]). Change from baseline to Week 8 also did not differ between groups on the MMSE (LSM [SE]: pimavanserin, 1.3 [0.15]; placebo, 1.2 [0.15]). Results were similar across patient NDD subgroups.

### Exploratory endpoints: suicidality and pimavanserin efficacy

**Fig. 2:**
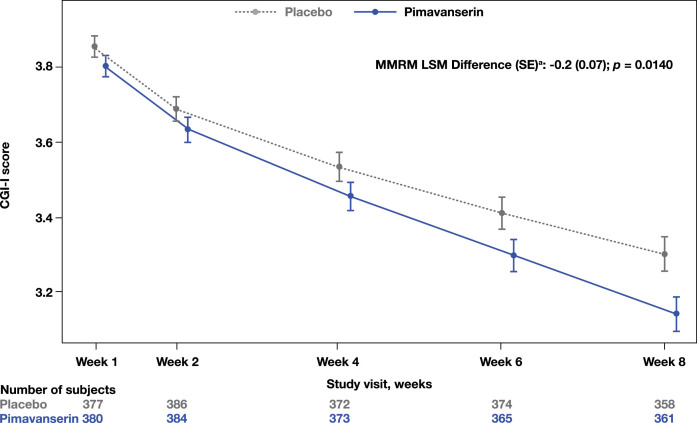
**CGI-I score throughout the study period.** ^a^LSM from MMRM with fixed categorical effects of region, planned treatment, visit, treatment-by-visit interaction, and fixed continuous covariates of baseline CGI-severity score and baseline CGI-severity score-by-visit interaction. CGI-I, Clinical Global Impression-Improvement; LSM, least square means; MMRM, mixed-effects model repeated measures.

**Fig. 3:**
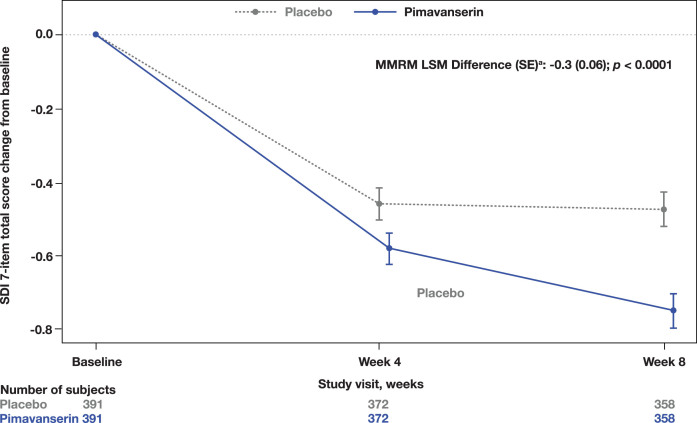
**Total score change from baseline on the SDI**. ^a^LSM from MMRM with fixed categorical effects of region, planned treatment, visit, treatment-by-visit interaction, and fixed continuous covariates of baseline SDI score and baseline SDI score-by-visit interaction. LSM, least square means; MMRM, mixed-effects model repeated measures; SDI, Sleep Disorders Inventory.

A significant improvement in the CGI-I was observed at Week 8 in the pimavanserin group compared to placebo (MMRM LSM difference (SE): −0.2 [0.07]; *p* = 0.0140; Fig. 2). Additionally, a significant improvement from baseline to Week 8 in the SDI was also observed (MMRM LSM difference [SE]: −0.3 [0.06]; *p* < 0.0001; Fig. 3). No significant differences were found in the CGI-S change from baseline to Week 8 (MMRM LSM difference [SE]: 0.0 [0.05], *p* = 0.3915) between groups or in the EQ-5D-5L visual analog scale (ANCOVA LSM; pimavanserin, 7.6; placebo, 6.4; *p* = 0.1943).

Four patients treated with pimavanserin (1.1%) and 1 patient treated with placebo (0.3%) reported postbaseline suicidal ideation. According to the C-SSRS assessment, no patients reported postbaseline incidence of suicidal behavior, self-injurious behavior with suicidal intent, or active suicidal ideation with the intent to act with or without a plan.

## DISCUSSION

Pimavanserin, the only FDA-approved antipsychotic to treat hallucinations and delusions associated with PDP (with or without dementia), is a well-established treatment with a favorable safety profile [1, 2]. There is a high degree of interest in further understanding the safety of pimavanserin, as many antipsychotics used off label often have significant and serious adverse effects, including risk of falls, parkinsonism, and death. [22]. This study served to further inform on the use and safety of pimavanserin in a placebo-controlled trial in a large population of patients with NDD who are sensitive to these potential adverse events associated with antipsychotic treatment. This trial demonstrated that pimavanserin is well tolerated in older adults and frail elderly patients with neuropsychiatric symptoms related to NDD, including Parkinson’s disease (with or without dementia) and other forms of dementia, including Alzheimer’s disease, dementia with Lewy bodies, and all-cause dementia. These results support the previously established safety profile of pimavanserin and further inform its use in patients with PDP [23–25].

A similar number of patients experienced TEAEs in both pimavanserin- (30.4%) and placebo-treated (29.3%) patients, and, importantly, mortality rates were also similar (0.5% per group); although, it should be noted that these TEAE incidence rates warrant continued safety monitoring. Furthermore, 92.6% of patients treated with pimavanserin completed the study, further indicating the favorable tolerability of pimavanserin in this patient population and highlighting a key strength of this study.

Treatment with pimavanserin was also not associated with cognitive decline (measured using the MMSE) or motor dysfunction (measured using the ESRS-A). This lack of cognitive or motor function-related side effects further highlight the safety and tolerability of pimavanserin. In addition, there were similar rates of suicidal ideation in both the pimavanserin (*n* = 4; 1.1%) and placebo (*n* = 1; 0.3%) groups. Notably, treatment with pimavanserin improved sleep quality, and improvement was also seen in neuropsychiatric symptoms in the CGI-I.

### Strengths and limitations

A key strength of this study is its large sample size in an elderly population compared to other studies that have assessed safety of pimavanserin [25, 26] and other commonly prescribed medications for psychosis in NDD [24, 27]. This study also evaluated patients across multiple clinical sites in several global regions. While changes in motor or cognitive function are commonly observed in patients following treatment with atypical antipsychotics, there were no differences between pimavanserin and placebo groups in this study [7–10], although these were secondary outcome measures. The American Geriatric Society 2019 Beers Criteria^®^ provides important guidance regarding the use of antipsychotics in elderly populations due to increased risk of falls and, thus, urge caution with prescribing these medications [28]; patients with dementia are also at risk of cerebrovascular accident, cognitive decline, and mortality. Thus, medications with an acceptable safety risk that are efficacious in patients with neuropsychiatric symptoms related to NDDs represent a key unmet need for this patient population.

The main limitations of this study are that the analysis was not powered to detect treatment differences within the different subgroups of the NDD identified, and no adjustments were made for multiplicity in the analyses. This study did not account for patients with an NDD who are < 60 years old, which may limit the generalizability of the results to a younger patient population. An additional limitation is the short duration of the study. Although a longer study duration would have considered late-emerging safety risks, pimavanserin has a long safety track record; additionally, given the patient population, 8-weeks was considered an appropriate study duration. Patients were also eligible to participate in a 52-week, open-label extension study. Finally, given that the clinical efficacy of pimavanserin was not the primary endpoint, this study does not provide direct clinical evidence to support the use of pimavanserin in this patient population. However, these results do provide important additional data on pimavanserin that support its use in patients with PDP (with or without dementia).

Together, these results highlight the manageable safety profile of pimavanserin in elderly patients with NDD, including Parkinson’s disease, and suggest an improvement in neuropsychiatric symptoms, consistent with previous findings.

### Conclusions

Pimavanserin was well tolerated and not associated with cognitive decline or motor dysfunction in patients with neuropsychiatric symptoms related to NDD. Safety data in this elderly population of patients with NDD were consistent with the well-characterized safety profile of pimavanserin and will help further inform its use to treat patients with PDP (with or without dementia).

## AUTHOR CONTRIBUTIONS

Gustavo Alva (Conceptualization; Formal analysis; Investigation; Writing – review & editing); Wiesław J. Cubała (Conceptualization; Formal analysis; Writing – review & editing); Ana Berrio (Conceptualization; Data curation; Formal analysis; Writing – review & editing); Bruce Coate (Conceptualization; Data curation; Formal analysis; Validation; Writing – review & editing); Victor Abler (Conceptualization; Formal analysis; Writing – review & editing); Sanjeev Pathak (Conceptualization; Formal analysis; Writing – review & editing).

## Data Availability

Clinical study documents are confidential and the property of Acadia. The protocol or statistical analysis plan is available upon request.
